# Massive Splenomegaly in Felty’s Syndrome: A Case Report

**DOI:** 10.7759/cureus.76021

**Published:** 2024-12-19

**Authors:** Laura N Soares, Juliana M Sternlicht, Nicole Jensen, Tomasia Jennyfer T de Aguiar, Marcelo Vincenzo Sarno Filho, Giovanna Caruso, Bruno Alvim C Araujo, Milton Glezer, Arnaldo Lichtenstein, Luiza Parra, Luis R Zogaib

**Affiliations:** 1 Internal Medicine, Hospital das Clínicas da Faculdade de Medicina da Universidade de São Paulo, São Paulo, BRA; 2 General Practice, Hospital das Clínicas da Faculdade de Medicina da Universidade de São Paulo, São Paulo, BRA; 3 Ophthalmology, Hospital das Clínicas da Faculdade de Medicina da Universidade de São Paulo, São Paulo, BRA

**Keywords:** chronic ulcer, diagnostic challenges, felty’s syndrome, neutropenia, rheumatoid arthritis, splenomegaly

## Abstract

Felty’s syndrome (FS) is a rare and complex condition most commonly seen as a complication of longstanding rheumatoid arthritis (RA), characterized by a triad of RA, splenomegaly, and neutropenia. Diagnosing FS can be challenging due to its diverse clinical presentations and overlap with other hematologic and autoimmune conditions. We report a 47-year-old male with a history of severe anemia, recurrent blood transfusions, and a chronic leg ulcer. In 2024, he presented with significant weight loss, polyarthralgia, and splenomegaly, prompting concern for a hematologic malignancy. Laboratory findings included elevated beta-2-microglobulin and positive antinuclear antibodies, raising suspicion of lymphoma or other malignancies. Extensive testing ruled out hematologic malignancies and schistosomiasis, and further investigation suggested FS. He was started on prednisone, filgrastim, and methotrexate, leading to substantial clinical improvement, including reduction of joint symptoms, spleen size, and improvement of the leg ulcer. This case highlights the diagnostic complexities of FS, especially in the absence of a prior RA diagnosis. FS should be considered in the differential diagnosis when encountering unexplained splenomegaly and neutropenia, even without a history of chronic RA. Early recognition and treatment can significantly improve patient outcomes. FS, though rare, should be considered in patients presenting with systemic symptoms, cytopenias, and splenomegaly. A thorough diagnostic workup is crucial to differentiate FS from other conditions, particularly hematologic malignancies. This case underscores the importance of prompt diagnosis and treatment to achieve favorable clinical outcomes.

## Introduction

Felty’s syndrome (FS) is a rare and potentially serious complication of rheumatoid arthritis (RA), characterized by the classic triad of rheumatoid arthritis, neutropenia, and splenomegaly [1]. It affects approximately 1% of patients with longstanding RA [2]. In recent years, the diagnosis of FS has become less common, potentially due to improved management of RA with therapies such as methotrexate and rituximab. Additionally, some cases of FS in the past may have been misdiagnosed as large granular lymphocyte (LGL) leukemia [3]. Despite these advancements, diagnosing FS remains challenging due to its rarity and the diverse range of clinical manifestations it can present with [1,3]. Here, we present a case of Felty’s syndrome with an atypical presentation that underscores the diagnostic complexities of this condition.

## Case presentation

A 47-year-old male was referred to the Internal Medicine Department of our tertiary care hospital in September 2024 for evaluation of a potential hematologic malignancy, such as splenic lymphoma or hairy cell leukemia, following transfer from another facility.

**Figure 1:**
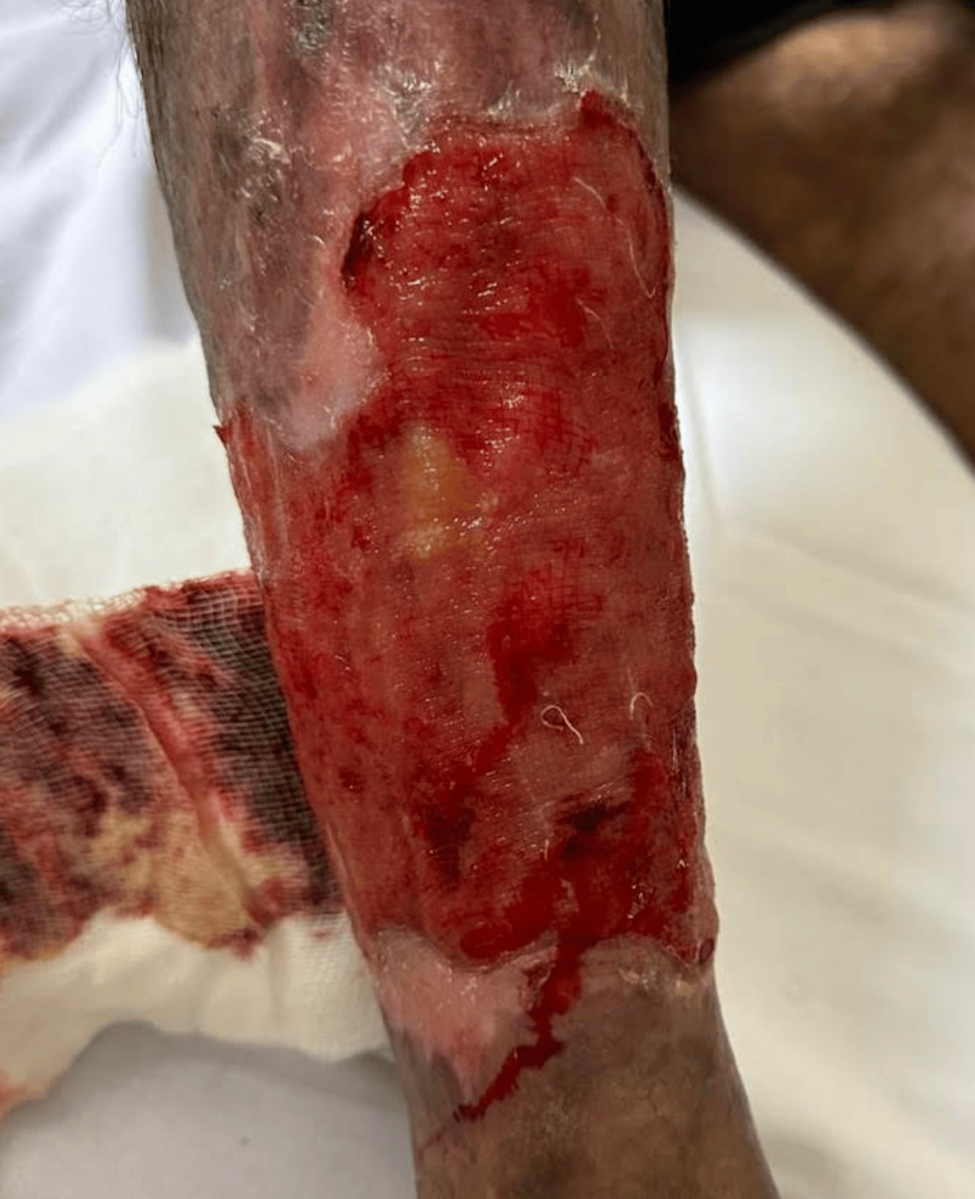
Left leg ulcer

The patient reported a history of severe anemia dating back to 2017, which necessitated intermittent blood transfusions despite ongoing iron supplementation. Additionally, he described a chronic ulcer on his right leg (Figure 1), which he attributed to a work-related trauma, and which had persisted since 2021. Despite local wound care, the ulcer progressively enlarged.

In early 2024, the patient experienced significant, unintended weight loss of approximately 30 kilograms in six months. In May 2024, he was hospitalized due to an infection in the ulcer. During this admission, he was diagnosed with severe anemia and thrombocytopenia. After discharge, he was referred for hematologic evaluation but was readmitted in September 2024 due to severe polyarthralgia, affecting multiple joints including the feet, knees, shoulders, elbows, wrists, and hands. The joint pain was exacerbated with movement but improved with rest. He also reported morning stiffness lasting approximately 10 minutes. On physical examination, the affected joints were warm, swollen, and erythematous. Due to the severity of his symptoms, he was hospitalized at another institution, where massive splenomegaly and neutropenia were identified, prompting his transfer for further hematologic assessment.

Although the patient did not initially report a history of joint pain, further inquiry revealed intermittent episodes of swelling and discomfort in both hands, which he attributed to his occupation as a metalworker.

Laboratory results from the referring institution revealed hemoglobin of 8.0 g/dL, a leukocyte count of 800/µL (with a differential of 416/µL neutrophils, 240/µL lymphocytes), and a platelet count of 175,000/µL. Additional findings included an elevated ferritin level of 1223 ng/mL, positive antinuclear antibodies with both homogeneous and speckled patterns (titers unspecified), a perinuclear anti-neutrophil cytoplasmic antibodies (P-ANCA) titer of 1:640, and low C3 levels (53 mg/dL), with normal C4 (418 mg/dL).

**Figure 2:**
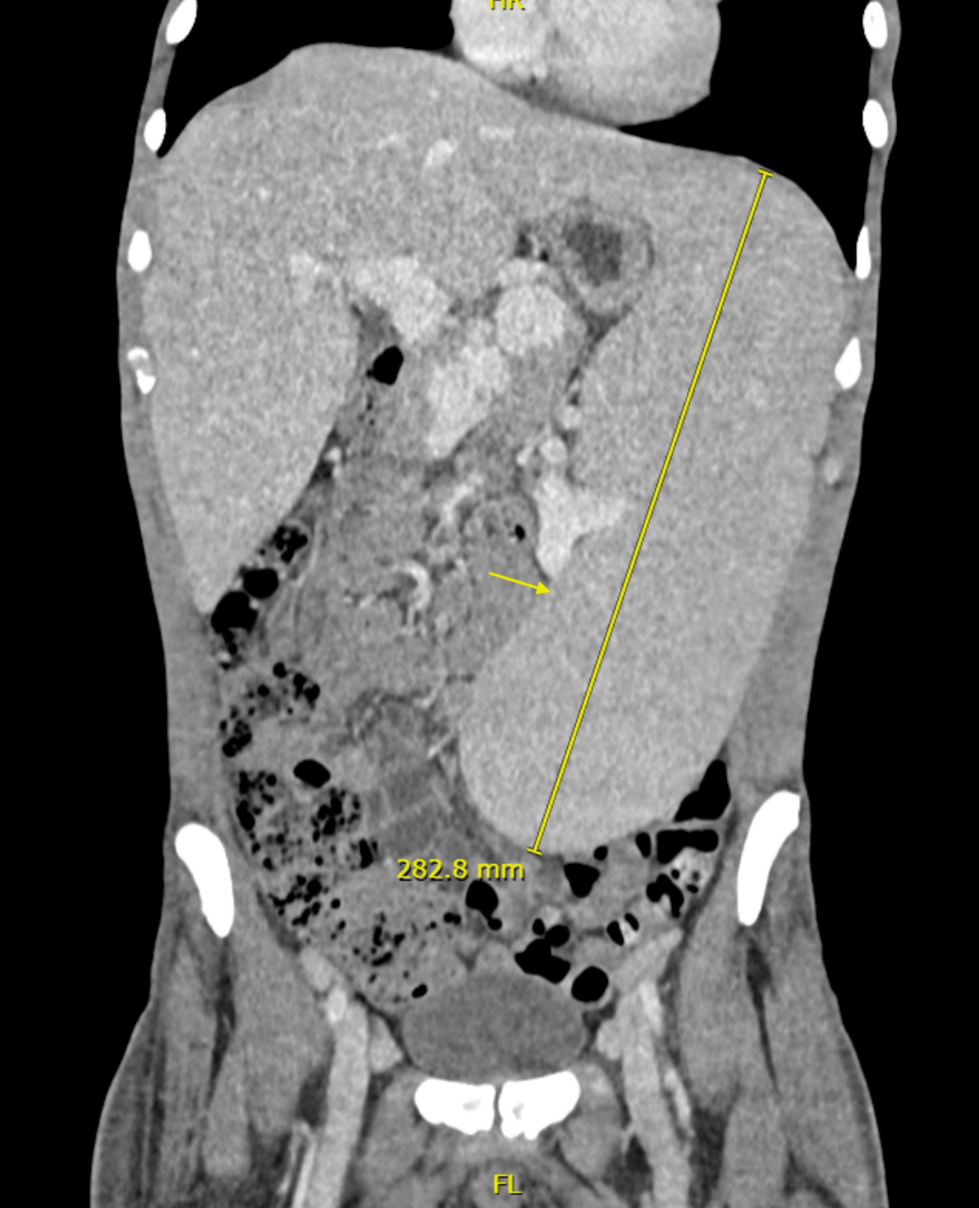
Computed Tomography of the abdomen Massive splenomegaly as indicated by the yellow arrow

Upon arrival at our hospital, a contrast-enhanced computed tomography (CT) scan revealed enlargement of axillary, iliac, and inguinal lymph nodes, as well as splenomegaly extending into the left iliac fossa (Figure 2). The spleen was homogeneous, and the liver was mildly enlarged but demonstrated regular margins.

**Figure 3:**
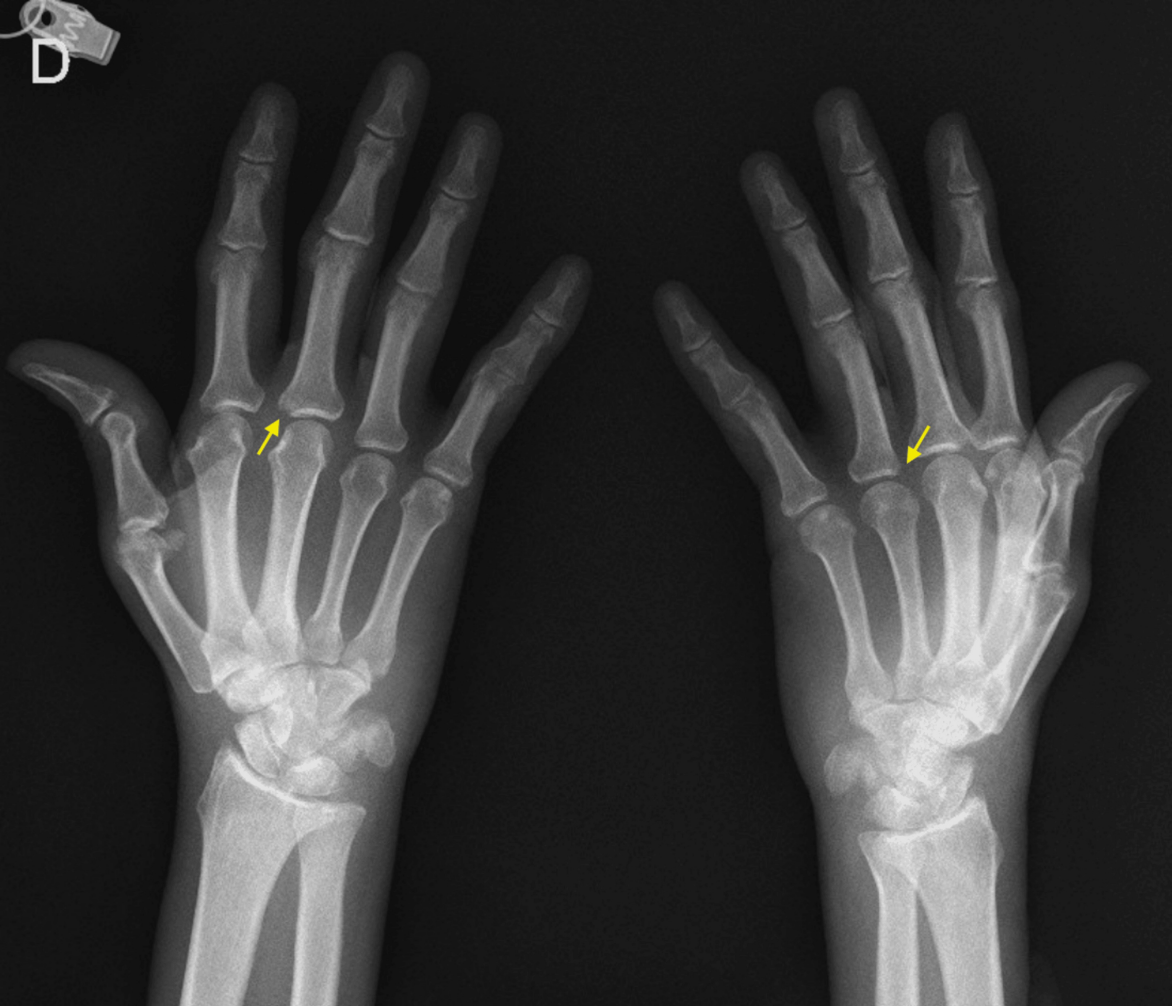
Hand X-ray Multiple marginal erosions as indicated by the yellow arrows

A hand X-ray revealed marginal erosions (Figure 3), and laboratory studies confirmed previous results, in addition to an elevated rheumatoid factor of 721 IU/mL and an elevated beta-2 microglobulin level of 7.67 mg/dL (Table 1).

**Table 1:** Laboratory findings P-ANCA: perinuclear anti-neutrophil cytoplasmic antibodies

Laboratory Test	Result	Reference Range
Hemoglobin (Hb)	8.0 g/dL	13.5–17.5 g/dL (male)
Leukocyte Count	800/µL	4,000–11,000/µL
Neutrophils	416/µL	2,000–7,500/µL
Lymphocytes	240/µL	1,000–4,800/µL
Platelet Count	175,000/µL	150,000–400,000/µL
Ferritin	1223 ng/mL	30–400 ng/mL
Antinuclear Antibodies (ANA)	Positive (homogeneous and speckled)	Negative (no titer specified)
P-ANCA	11:40	Negative (<1:40)
C3	53 mg/dL	90–180 mg/dL
C4	418 mg/dL	10–40 mg/dL
Rheumatoid Factor	721 IU/mL	<20 IU/mL
Beta-2 Microglobulin	7.67 mg/dL	1.0–2.5 mg/dL
Bone Marrow Studies	Reticulocytosis	Normal
HIV Serology	Negative	Negative
Hepatitis B Serology	Negative	Negative
Hepatitis C Serology	Negative	Negative
Schistosomiasis Serology	Positive	Negative

Based on these findings, we initially considered a diagnosis of RA with Felty syndrome. However, the pronounced splenomegaly and the absence of a prolonged history of RA prompted further diagnostic scrutiny.

To rule out hematologic malignancies, we performed a biopsy of the leg ulcer, which revealed no evidence of granulomas or vasculitis. A biopsy of an axillary lymph node demonstrated only reactive lymphadenopathy without signs of granulomas or malignancy. Serum immunophenotyping studies returned normal results, and bone marrow studies revealed only reticulocytosis. Infectious disease testing for HIV, hepatitis B, and hepatitis C was negative. Notably, serology for schistosomiasis was positive. The patient had been born and lived in a region of Brazil with a high prevalence of schistosomiasis until the age of 12. However, CT imaging showed no evidence of hepatosplenic involvement by schistosomiasis, such as portal hypertension or enlargement of the hepatic fissures.

Given the lack of other differential diagnoses and the strong possibility of Felty syndrome, on September 28th, we initiated treatment targeting this condition. The patient was started on a regimen of prednisone, filgrastim, and methotrexate (initial dose of 10 mg per week). A hemogram performed on September 30th revealed a leukocyte count of 730/µL and 390/µL neutrophils. By the time of discharge on September 20th, the leukocyte count had increased to 2520/µL, with 1640/µL neutrophils. The patient reported substantial improvement in joint pain, with a reduction in swelling and less morning stiffness.

Two weeks post-discharge, the patient returned for follow-up and continued to show positive progress. His white blood cell count remained elevated, and nearly complete resolution of his hand arthritis was noted. Physical examination revealed a reduction in spleen size and an improved appearance of the leg ulcer. The patient was subsequently referred to the Rheumatology team at our hospital, where he continues to be followed.

## Discussion

FS presents a diagnostic challenge due to its rarity, lack of pathognomonic signs, and absence of a definitive diagnostic test. Its clinical features often overlap with those of hematologic malignancies, complicating the diagnostic process. The case presented here was particularly difficult, as the patient's clinical presentation was atypical, and he did not have a prior diagnosis of RA, which is typically associated with FS [1].

The evaluation of splenomegaly and cytopenias involves a broad differential diagnosis, including infections, malignancies, storage diseases, congestive causes, and inflammatory disorders [4]. Massive splenomegaly, however, is a relatively rare finding in these conditions. Hairy cell leukemia is a notable cause of massive splenomegaly accompanied by cytopenias, as is splenic lymphoma [5]. Our patient presented with an elevated beta-2-microglobulin level, a biomarker commonly associated with lymphoid malignancies due to its release from white blood cell membrane turnover [6]. Additionally, he had significant unintentional weight loss, another feature suggestive of a potential hematologic malignancy. However, after a thorough evaluation- including blood tests, lymph node biopsy, serum immunophenotyping, and bone marrow studies- we did not identify any monoclonal proliferations or abnormalities indicative of hematologic malignancy. It is worth noting, however, that beta-2-microglobulin levels can also be elevated in autoimmune conditions such as FS. In fact, a study by Falus et al. (1983) demonstrated significantly higher beta-2-microglobulin levels in FS patients compared to those with joint-restricted RA, which further supports this biomarker’s role in FS diagnosis [7].

Schistosomiasis, another potential cause of splenomegaly and cytopenias, must also be considered in the differential diagnosis, particularly in endemic areas. Our patient’s positive serology for schistosomiasis raised this concern. Although serological tests are often used in diagnosing active schistosomiasis, they may not be as informative in patients from endemic regions. Stool examination using the Kato-Katz technique can identify schistosomal eggs, though its sensitivity is limited, as it depends on the presence of active egg excretion [8]. Conversely, CT imaging, with a sensitivity of 90%, is highly effective for detecting signs of portal hypertension, making it a valuable tool in investigating schistosomiasis. In our patient, the CT scan did not show evidence of portal hypertension, which helped exclude schistosomiasis as the cause of splenomegaly [9].

In the context of FS, while splenomegaly is a nearly ubiquitous finding, massive splenomegaly is not commonly described in the literature. A 1997 study by Sienknecht et al. did report spleen weights ranging from 210 to 1650 grams in patients with FS who underwent splenectomy, potentially correlating with the large splenic size observed in our patient on physical examination [10].

Historically, FS is recognized as a complication of longstanding, severe RA. Campion (1990) reported a mean interval of 13.7 years between the diagnosis of RA and the onset of FS in male patients [11]. In contrast, our patient exhibited a severe form of FS without a prior diagnosis of RA, and only subacute arthritis was present. Although exceedingly rare, cases of FS without overt arthritis have been documented in the literature, further emphasizing the heterogeneous nature of this condition [12].

## Conclusions

FS remains a rare and diagnostically challenging condition, particularly when its presentation diverges from typical patterns, as seen in this case. While FS is most commonly associated with longstanding RA, our patient presented with a severe form of FS without a prior diagnosis of RA, highlighting the heterogeneous nature of the syndrome. The case underscores the importance of a comprehensive diagnostic approach when dealing with unexplained splenomegaly, cytopenias, and systemic symptoms, as these can overlap with a wide range of hematologic, infectious, and autoimmune conditions. In this patient, the combination of splenomegaly, neutropenia, and elevated beta-2-microglobulin, along with other findings such as a positive serology for schistosomiasis, required the exclusion of several differential diagnoses. The absence of malignancy, coupled with the patient’s clinical improvement following treatment with prednisone, filgrastim, and methotrexate, ultimately confirmed FS. This case illustrates the importance of considering FS in the differential diagnosis even in the absence of a prior RA diagnosis, particularly in patients presenting with unexplained systemic symptoms and cytopenias. Early recognition and appropriate treatment can lead to significant clinical improvement, as demonstrated in this case. Thus, clinicians should maintain a high index of suspicion for FS in patients presenting with unexplained systemic symptoms, splenomegaly, and neutropenia, especially when classic findings of RA are absent or not well established.
